# Prediction models for clustered data with informative priors for the random effects: a simulation study

**DOI:** 10.1186/s12874-018-0543-5

**Published:** 2018-08-06

**Authors:** Haifang Ni, Rolf H. H. Groenwold, Mirjam Nielen, Irene Klugkist

**Affiliations:** 1 Department of Methodology and Statistics, Faculty of Social and Behavioral Sciences, Utrecht University, Utrecht, The Netherlands; 2 Department of Farm Animal Health, Faculty of Veterinary Medicine, Utrecht University, Utrecht, The Netherlands; 3 Department of Epidemiology, Julius Center for Health Sciences and Primary Care, University Medical Center Utrecht, Utrecht, The Netherlands; 4 Department of Clinical Epidemiology, Leiden University Medical Centre, Leiden, The Netherlands; 5 Research Methodology, Measurement and Data Analysis of Behavioral, Management and Social Sciences, Twente University, Enschede, The Netherlands

**Keywords:** Random effects prediction model, Clustered data, Informative priors for the random effects, Expert knowledge, Truncated distribution

## Abstract

**Background:**

Random effects modelling is routinely used in clustered data, but for prediction models, random effects are commonly substituted with the mean zero after model development. In this study, we proposed a novel approach of including prior knowledge through the random effects distribution and investigated to what extent this could improve the predictive performance.

**Methods:**

Data were simulated on the basis of a random effects logistic regression model. Five prediction models were specified: a frequentist model that set the random effects to zero for all new clusters, a Bayesian model with weakly informative priors for the random effects of new clusters, Bayesian models with expert opinion incorporated into low informative, medium informative and highly informative priors for the random effects. Expert opinion at the cluster level was elicited in the form of a truncated area of the random effects distribution. The predictive performance of the five models was assessed. In addition, impact of suboptimal expert opinion that deviated from the true quantity as well as including expert opinion by means of a categorical variable in the frequentist approach were explored. The five models were further investigated in various sensitivity analyses.

**Results:**

The Bayesian prediction model using weakly informative priors for the random effects showed similar results to the frequentist model. Bayesian prediction models using expert opinion as informative priors showed smaller Brier scores, better overall discrimination and calibration, as well as better within cluster calibration. Results also indicated that incorporation of more precise expert opinion led to better predictions. Predictive performance from the frequentist models with expert opinion incorporated as categorical variable showed similar patterns as the Bayesian models with informative priors. When suboptimal expert opinion was used as prior information, results indicated that prediction still improved in certain settings.

**Conclusions:**

The prediction models that incorporated cluster level information showed better performance than the models that did not. The Bayesian prediction models we proposed, with cluster specific expert opinion incorporated as priors for the random effects showed better predictive ability in new data, compared to the frequentist method that replaced random effects with zero after model development.

**Electronic supplementary material:**

The online version of this article (10.1186/s12874-018-0543-5) contains supplementary material, which is available to authorized users.

## Background

In many medical areas, prediction models are used to support clinical practice [1].

When study data collected for the development of a prediction model are clustered e.g., patients are registered with the same general practitioner or farm animals live in the same herd, there is often within cluster dependency. It is suggested that the clustering structure should be taken into account in the development of a prediction model, in order to produce unbiased model parameter estimates [2], whereas regression methods that assume independence between subjects are inappropriate. In such situations, random effects regression analysis can be a viable alternative, as it parameterizes the cluster level heterogeneity by means of random effects, and allows predictions to be made at the subject level [3].

Surprisingly, despite the routine use of random effects regression modelling in etiological or intervention research, this approach is hardly seen in prediction research [4]. This is probably because generalization of the random effects model is not straightforward [5], as the latent random coefficient of a new cluster is considered unknown. In existing clinical applications, e.g., Van der Drift et al. [6], the random effects were removed from the model after selection of predictors at the model development phase. This is equivalent to setting the random effects for all new clusters to zero. By doing so, the prediction model simply ignores the clustering structure in new data, which may lead to a loss of prediction accuracy [2].

Alternatively, one could maintain and estimate the random effects for new clusters by incorporating external cluster level information into the prediction model. In medical practice for instance, some hospitals are better at treating a certain disease than other hospitals due to hospital specific characteristics. An expert may be able to provide such additional information about the hospitals. Methods for eliciting information from experts can be found in literature such as Spiegelhalter et al. [7] and O’Hagan et al. [8]. Incorporation of expert knowledge into the data analysis can easily be done under the Bayesian framework. In this paper, we propose a new approach that includes cluster level expert knowledge as prior evidence for the random effects in a prediction model and investigate the benefit of this approach in the setting of new clustered data.

The paper is organized as follows: in the Methods section, we first review how one can develop and apply a prediction model either in a frequentist or in a Bayesian way. We then propose our approach of incorporating expert opinion into the prediction model. Description of the simulation studies is provided afterwards, followed by the Results section. The paper concludes with a discussion of results and implications for future research.

## Methods

### Estimation at model development phase

At the model development phase, data containing measures of the predictor(s) and outcome of interest are collected for the purpose of estimating the model parameters. In empirical applications, selection of relevant predictor(s) is often performed first. In this study, we assume that relevant predictors were selected already and directly focus on parameter estimation.

We consider a simple logistic regression model with one predictor measured on the subject level and random effects at the cluster level. Let *x*_*ij*_ be the predictor and *y*_*ij*_ be the observed binary outcome of subject *i* (*i* = 1, …, *n*_*j*_) from cluster *j* (*j* = 1, …, *J*), and *p*_*ij*_ = *p*(*y*_*ij*_ = 1) be the latent underlying risk for the observed binary outcome. A random effects logistic regression model can be expressed as:

2.1 $$ {\displaystyle \begin{array}{c} logit\ \left({p}_{ij}\right)={\beta}_0+{\beta}_1{x}_{ij}+{u}_j\\ {}{u}_j\sim N\left(0,{\sigma}_u^2\right),\end{array}} $$

where the logit (i.e., log-odds) of the latent underlying risk of the outcome *logit*(*p*_*ij*_) is equivalent to the linear predictor *LP*_*ij*_ = *β*_0_ + *β*_1_*x*_*ij*_ + *u*_*j*_. The model can alternatively be written in the form of:

2.2 $$ {p}_{ij}=\frac{1}{1+\exp \left(-{LP}_{ij}\right)}. $$

The linear predictor consists of the regression parameter *β*_1_ for the predictor, the average (fixed) intercept *β*_0_ and the cluster specific random effect *u*_*j*_. The random effects are assumed to have a normal distribution with mean zero and variance ${\sigma}_u^2$.

### Frequentist estimation

Within the frequentist approach, parameters of a logistic regression prediction model are estimated via maximum likelihood (ML). In our study, functions from the R package ‘lme4’ were used [9].

### Bayesian estimation

Within the Bayesian framework, parameters are expressed in the form of distributions rather than fixed values. Before observing the data, prior distributions that contain the plausible values for the model parameters need to be specified [7]. The prior distributions are subsequently updated with observed data, resulting in posterior distributions. The posterior can be derived either analytically or by sampling methods. In this study, Markov chain Monte Carlo (MCMC) sampling was used.

Prior distributions needed to be specified for parameters *β*_0_, *β*_1_ and ${\sigma}_u^2$ in model (2.1). Priors for the regression parameters *β*_0_ and *β*_1_ were assumed to be normally distributed, and prior for the variance ${\sigma}_u^2$ had an inverse gamma distribution. When there is no a priori evidence available, one often uses weakly informative priors. A common choice for a normal distribution is to fix the mean at zero and take a large variance (here we used 1000 for the variance). For an inverse gamma distribution, small values are often assigned to the hyperparameters (here we used 0.001 for both hyperparameters).

2.3 $$ {\displaystyle \begin{array}{c}\beta \alpha \sim N\ \left(0,1000\right)\ \left(\mathrm{for}\ \alpha =0,1\right),\\ {}{\sigma}_u^2\sim Inv- gamma\ \left(0.001,0.001\right).\end{array}} $$

Estimates for the parameters of interest are provided by the MCMC samples from the posterior distribution after convergence. In this study, we used OpenBUGS (via R package ‘BRugs’ [10]) to carry out the Bayesian analyses.

### Prediction in new clusters

Let *x*_*sc*_ be the predictor, *y*_*sc*_ be the observed binary outcome and *p*_*sc*_ be the latent underlying risk of the observed outcome for subject *s* (*s* = 1, …, *n*_*c*_) from new cluster *c* (*c* = 1, …, *C*)*.* The prediction model developed and estimated from model development data is applied to calculate the risk of outcome for each subject in new clusters. The predicted risk of outcome ${\widehat{p}}_{\mathrm{sc}}$ is compared to the true latent underlying risk *p*_*sc*_ and the observed outcome *y*_*sc*_ for the evaluation of the predictive performance.

### Frequentist prediction

In the frequentist approach, prediction for new clusters is usually based on a model where point estimates for the regression parameters are incorporated and the random effect term is substituted with mean 0 (i.e., removed). This leads to the predicted linear predictor.

2.4 $$ {\widehat{LP}}_{sc}^{ML}={\widehat{\beta}}_0^{ML}+{\widehat{\beta}}_1^{ML}{x}_{sc}, $$

where ${\widehat{\beta}}_0^{ML}$ and ${\widehat{\beta}}_1^{ML}$ are the estimated regression coefficients using maximum likelihood estimation and *x*_*sc*_ contains values of the predictor from subjects in new clusters. Accordingly, the predicted risk for the binary outcome in new clusters can be written as

2.5 $$ {\widehat{p}}_{\mathrm{sc}}=\frac{1}{1+\exp \left(-{\widehat{LP}}_{sc}^{ML}\right)}. $$

### Bayesian prediction

In the Bayesian approach, by MCMC sampling, we obtain the posterior distribution for the parameters *β*_0_, *β*_1_ and ${\sigma}_u^2$. In this study, instead of using summarized point estimates, the full posterior for the parameters is exploited for prediction. To explain the Bayesian prediction, consider Table 1 in which a small part of the MCMC output is listed.

**Table 1:** An example of using posterior samples from model development data analysis for prediction in a new cluster

Posterior from model development data					Prediction for new cluster *c*				
Iteration					Subject 1		**…**	Subject *n*_*c*_	
*k*	${\overset{\sim }{\beta}}_0^{(k)}$	${\overset{\sim }{\beta}}_1^{(k)}$	${{\overset{\sim }{\sigma}}_u^2}^{(k)}$	${\widehat{u}}_c^{(k)}$ ^a^	*x*_1 *c*_	${\widehat{p}}_{1c}^{(k)}$ ^b^	**…**	${x}_{n_cc}$	${\widehat{p}}_{n_cc}^{(k)}$ ^b^
**.**	**.**	**.**	**.**	**.**	**.**	**.**	**.**	**.**	**.**
**.**	**.**	**.**	**.**	**.**	**.**	**.**	**.**	**.**	**.**
5001	−1.35	1.07	1.17	.50	1.11	.58	**…**	−.46	.21
5011	−1.24	1.08	.88	−1.89	1.11	.13	**…**	−.46	.03
5021	−1.36	1.18	1.28	−.06	1.11	.47	**…**	−.46	.12
5031	−1.31	1.05	.98	−.64	1.11	.31	**…**	−.46	.08
5041	−.94	.98	1.37	.26	1.11	.60	**…**	−.46	.24
**.**	**.**	**.**	**.**	**.**	**.**	**.**	**.**	**.**	**.**
**.**	**.**	**.**	**.**	**.**	**.**	**.**	**.**	**.**	**.**
Median						.52			.15

^a^random effect sampled from the normal distribution $N\left(0,{{\overset{\sim }{\sigma}}_u^2}^{(k)}\right)$

^b^predicted risk calculated by ${\widehat{p}}_{\mathrm{sc}}^{(k)}=\frac{1}{1+\exp \left(-{\overset{\sim }{\beta}}_0^{(k)}+{\overset{\sim }{\beta}}_1^{(k)}{x}_{sc}+{\widehat{u}}_c^{(k)}\right)}$

The sampled values for parameters from iteration *k*, denoted by ${\overset{\sim }{\beta}}_0^{(k)}$,${\overset{\sim }{\beta}}_1^{(k)}$ and ${{\overset{\sim }{\sigma}}_u^2}^{(k)}$, are presented in the left hand part of the table. Predictions made for subjects in a new cluster can be found in the right hand part of the table. As the model development clusters and the new clusters are assumed to be exchangeable, i.e., originated from the same metapopulation, random effects for all clusters are assumed to have the same normal distribution $N\left(0,{\sigma}_u^2\right)$. For new cluster *c*, in each iteration, the predicted random effect ${\widehat{u}}_c^{(k)}$ can be drawn from distribution$N\left(0,{{\overset{\sim }{\sigma}}_u^2}^{(k)}\right)$. For each subject *s* (*s* = 1, …., *n*_*c*_) from cluster *c*, the estimated linear predictor is hence

2.6 $$ {\widehat{LP}}_{sc}^{(k)}={\overset{\sim }{\beta}}_0^{(k)}+{\overset{\sim }{\beta}}_1^{(k)}{x}_{sc}+{\widehat{u}}_c^{(k)}, $$

and the predicted risk can be obtained by.

2.7 $$ {\widehat{p}}_{\mathrm{sc}}^{(k)}=\frac{1}{1+\exp \left(-{\widehat{LP}}_{sc}^{(k)}\right)} $$

Eventually, a predicted risk distribution based on all K iterations is available for each subject. In this paper, in order to compare the results between the Bayesian models and the frequentist model, we used the median of the predicted risk distribution as the summarized predicted risk ${\widehat{p}}_{\mathrm{sc}}$, resulting in a single estimate per subject.

By applying the Bayesian approach, we can maintain the random effect term in the prediction model, which takes uncertainty due to variance between clusters into account. To improve the predictive ability of the Bayesian random effects model, one may include cluster specific expert knowledge as prior for the random effects on new clusters. Demonstration of how this prior knowledge was incorporated into a Bayesian prediction model can be found in the following section.

### Bayesian approach with informative priors

In the previous section, for each new cluster *c* in each iteration *k*, the predicted random effect ${\widehat{u}}_c^{(k)}$ was sampled from the entire random effects distribution $N\left(0,{{\overset{\sim }{\sigma}}_u^2}^{(k)}\right)$. This would add more uncertainty to the prediction compared to the frequentist model that substituted the random effects with the mean 0. However, if there is information available about the position of a new cluster relative to other clusters, we may sample a value for ${\widehat{u}}_c^{(k)}$ from part of the distribution rather than the whole distribution.

**Fig. 1:**
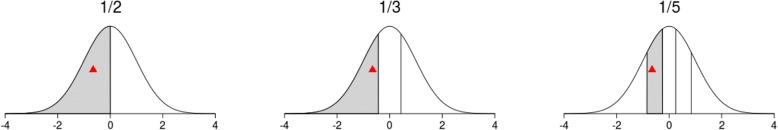
The random effects distribution divided into multiple truncated areas of equal proportions in 3 different scales. A truncated area contains either half, one third, or one fifth of the distribution. Based on elicited expert knowledge for each cluster, a particular truncated area from each scale is chosen and used as prior distribution for the random effect of the cluster. We considered a prior distribution that contains 1/2 of the distribution as low informative, 1/3 as medium informative, and 1/5 as highly informative

Consider again the example of hospitals where some hospitals are known to be better at treating a particular disease than others. When we have no clue about the relative risk of death for a disease from a particular hospital regarding other hospitals, we sample a random effect for the hospital from the entire random effects distribution. However, if an expert is capable of using hospital level information to judge whether a hospital would provide below or above average chance of survival, we could sample the random effect from only the lower or upper half of the distribution [11]. Suppose the expert says the hospital will provide a below average probability of survival, the random effect will accordingly be sampled from the lower half of the distribution (see the first plot in Fig. 1). Further, if the expert is more precise about the relative position of the hospital with regard to other hospitals, the random effect can also be drawn from a smaller area, such as one third or one fifth of the distribution (see the second and third plots in Fig. 1).

It is expected that more precise prior knowledge leads to better predictions, under the assumption that an expert provides information that matches the true value. It is however likely that experts sometimes provide suboptimal judgments that deviate from the true quantity. Incorporation of discrepant expert opinion into the prediction model was therefore investigated as well. All investigations were done through simulations. Details of the simulation studies are presented in the next section.

## The simulation studies

### Data generation

One set of data containing 5000 subjects (*n* = 5000) was generated for model development. The number of clusters was set to 50, and the number of subjects per cluster was 100. Each cluster consisted of equal numbers of subjects. For each subject, one continuous predictor was sampled from the normal distribution *N*(0, 1) and allocated to the subjects in all further analyses. The true value for the regression parameter *β*_1_ was set to 1.5. The random effects for clusters were sampled from a normal distribution with cluster variance 0.822. This value corresponds to an intraclass correlation coefficient (ICC) of 0.20, calculated from the formula ${\sigma}_u^2/\left({\sigma}_u^2+{\pi}^2/3\right)$ where the error variance has a fixed value *π*^2^/3 in a logistic regression model [3]. The latent underlying risk of the outcome for each subject was calculated by computing the linear predictor. The observed outcome was subsequently sampled from a Bernoulli distribution using its underlying risk. By adjusting the value of the fixed intercept *β*_0_, we set the overall prevalence of the dataset approximately to 50%. Finally, one new dataset was simulated by the exact same setting and used to evaluate the different prediction models. The simulation study thus contained one dataset for model development and one dataset for prediction. The R code for data simulation is provided in Additional file 2: Appendix B. It is worth noting that each time if we replicate a simulation study using the same settings with a different seed, we get different samples for the model development and prediction datasets, as there is randomness involved in the sampling process. Comparisons of the relative performance between models were hence carried out only within the same sets of development and prediction data.

### Analysis of simulated data

Five models were estimated using model development data and applied to predict in new data. In the frequentist model (denoted *FREQ*), ML estimates of the regression parameters ${\widehat{\beta}}_0^{ML}$ and ${\widehat{\beta}}_1^{ML}$ were incorporated, and the random effect term was replaced with 0. Within the Bayesian approach, a prediction model using weakly informative priors for the random effects was first specified and denoted *BAYES.WI*. Three more prediction models were subsequently constructed where cluster specific expert judgments were incorporated as prior information for the random effects. Optimal expert judgment was defined as choosing the truncated area from the random effects distribution which contained the true value of the random effect. Three scales were specified for the random effects distribution on the basis of varying degrees of precision of the expert opinion. In each scale, the distribution was divided into equal sized truncated areas (i.e., equal proportions). The model that used low informative priors which contained half of the distribution was denoted *BAYES.LI.* Similarly, the model that used medium informative priors which contained one third of the distribution was denoted *BAYES.MI*, and the model that used highly informative priors which contained one fifth of the distribution was denoted *BAYES.HI*. For each Bayesian prediction model, two posterior chains were sampled and thinned by the interval 10 (i.e., taking every 10th observation). Within each chain 100 samples were saved after convergence was reached, leading to 200 posterior samples in total for prediction in each subject from the new clusters.

### Discrepant expert opinion

Impact of including expert opinion that deviated from the true random effect value as prior information was explored in models *BAYES.LI*, *BAYES.MI* and *BAYES.HI*. We defined discrepant expert judgment as selecting a truncated area that was next to the ‘correct’ area that contained the true value of the random effect. In the *BAYES.LI* condition, it was straightforward, since the random effects distribution was divided into two equal sized areas. When the true value of the random effect was located in one area, the other area would be chosen. However, in the *BAYES.MI* and *BAYES.HI* conditions where the random effects distribution was divided into more than two equal sized areas, choosing a truncated area that was next to the ‘correct’ area indicated that the discrepant expert opinion was still relatively close to the true value (see Fig. 1). Suppose the true value was at the middle 1/5 truncated area of the distribution, the discrepant expert opinion would be selecting the second 1/5 or the fourth 1/5 truncated area from the left hand side. In other words, discrepant expert opinion with more precision (i.e., 1/3, 1/5) was less off from the true value in comparison to the discrepant expert opinion with least precision (1/2). The percentage of new clusters that incorporated discrepant expert opinion was set to 10, 30% or 50%.

### Expert opinion as categorical variable in the frequentist approach

In principle, one could also include prior knowledge in the frequentist model. We performed simulations where optimal expert opinion was incorporated as a fixed effect in the frequentist model in the default scenario. First, in the phase of model development, the true random effects were used to create the additional predictor representing the prior knowledge. For instance, when the true random effect for a specific cluster was among the upper half of the true random effects distribution, expert opinion for this cluster was then coded into 1 when the lower half was the reference category (coded 0). Likewise, expert opinion was coded using a categorical variable with 3 or 5 levels by placing the true random effects for the model development clusters in the correct tertiles and quintiles, with the second tertile and the third quintile as the reference category respectively. The frequentist models with inclusion of expert opinion coded into 2, 3, and 5 categories were denoted *FREQ.2*, *FREQ.3* and *FREQ.5* respectively.

The resulting prediction models with expert opinion were used to predict the outcomes in the new clusters. Again, the expert knowledge (i.e., the scores on the categorical variable) for each new cluster was obtained by placing the true random effect for the new cluster in the correct quantiles of the true random effects for the model development clusters.

### Assessment and comparison of model performance

The predictive ability of the models was assessed by Brier scores, model discrimination and calibration. Brier score was computed as the mean of the squared difference between the observed binary outcomes and the predicted risks. Discriminative ability was assessed by the concordance index (C-index) which equals the area under the ROC curve. Model calibration was evaluated using the calibration slope. The ideal value for the calibration slope is 1, which represents perfect prediction. The further the calibration slope deviates from 1, the worse the model is calibrated [1]. Since in the simulation research, true latent underlying risks of outcome are available, the calibration was computed as the agreement between predicted risks and true risks. The calibration slope was the linear regression coefficient for the predicted risk as the independent variable, and the true risk as the dependent variable. It is worth noting that in empirical data, true risks are not available. Model calibration can hence be assessed by using the observed binary outcome as the dependent variable and the estimated linear predictor as the independent variable in a logistic regression analysis [1, 12]. Brier scores were computed at the subject level (overall) for all models. Model discrimination and calibration were measured both at subject level (overall) and cluster level (within cluster). The within cluster measures were summarized in mean and standard deviation over all clusters. Calibration of the models was further visualized in calibration plots where the predicted risks of outcome were plotted against the true risks.

### Sensitivity analyses

In order to check the influence of prevalence, ICC, sample size and strength of the predictor on prediction, eight sensitivity analyses were carried out. Each sensitivity analysis consisted of one new simulation study that had default simulation settings except for the specific feature that was investigated. Impact of the between cluster variance was evaluated by comparing the default ICC value 0.20 to 0.05 and 0.50. Impact of the prevalence was evaluated by comparing the default prevalence value 50 to 10% and 25%. To examine the effect of smaller sample sizes on prediction, we reduced in one sensitivity analysis the number of clusters, resulting in *n* = 2000 subjects in total ( *J* = *S* = 20, *n*_*j*_ = *n*_*c*_ = 100), and in another sensitivity analysis the number of subjects per cluster, resulting in *n* = 1000 subjects in total ( *J* = *S* = 50, *n*_*j*_ = *n*_*c*_ = 20). Finally, by changing the value for the model parameter *β*_1_ from 1.5 (default) to 0.5 and 3.0, we explored the influence of a weaker or a stronger subject level predictor.

## Results

**Fig. 2:**
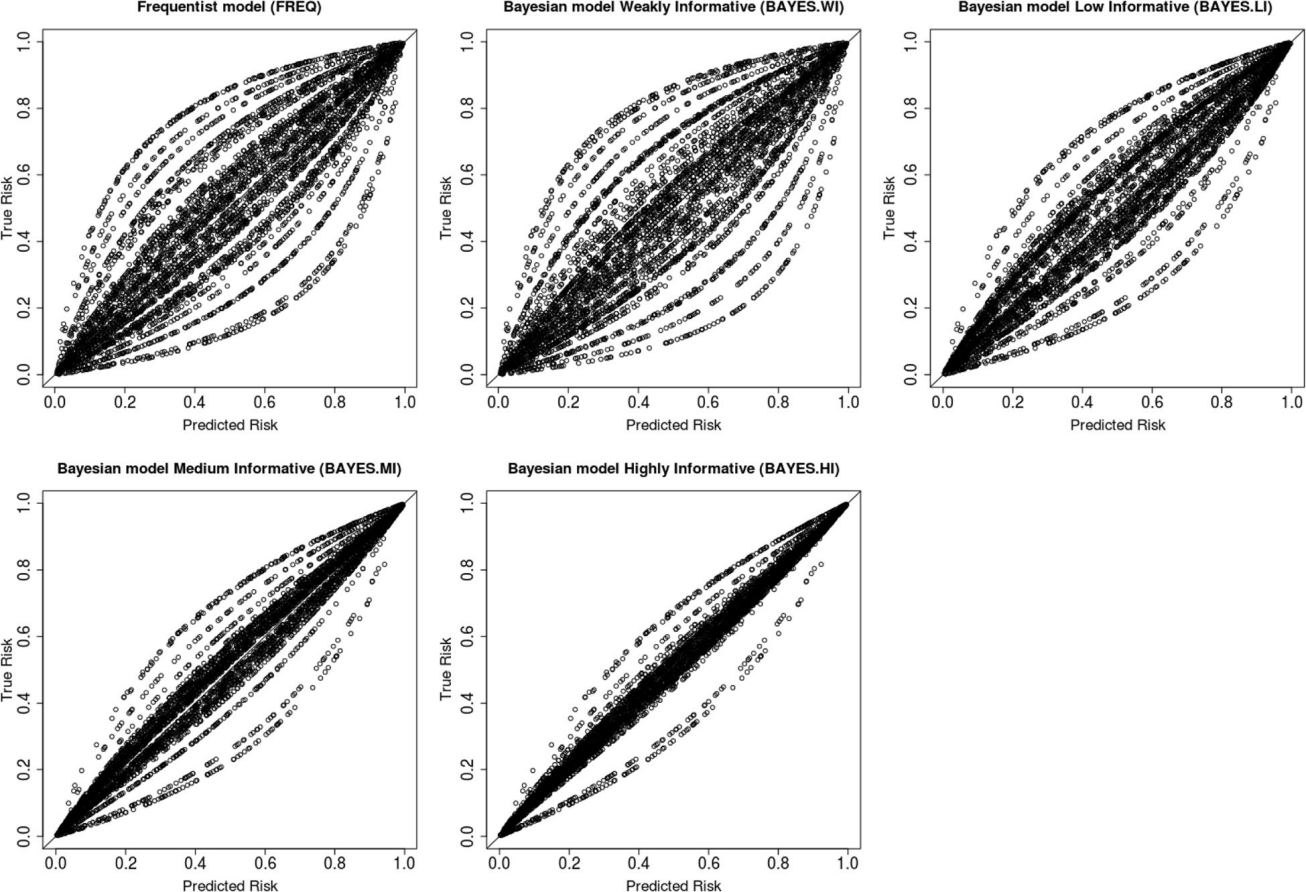
Calibration plots for the five prediction models. Predicted risks are plotted against the true latent underlying risks for 5000 subjects from 50 equal sized clusters. The diagonal indicates the line of identity (i.e., predicted risks are equivalent to the true risks). Each dot represents a subject, and each line formed by the dots represents a cluster

As can be seen in Table 2, the frequentist model and the Bayesian model without prior information showed, as expected, approximately the same Brier scores, similar discrimination and calibration at the overall as well as the cluster level. The Bayesian models with informative priors showed smaller Brier scores and larger overall C-indexes. The increasing C-indexes also revealed a positive relation between the precision of expert opinion and the overall discrimination. Further, difference in overall calibration slopes suggested that the Bayesian models with informative priors had better overall calibration. This can also be inspected in the calibration plots in Fig. 2, where the predicted risks were plotted against the true latent underlying risks. Smaller difference between the predicted and true risks can be seen for the Bayesian models with informative priors, as the calibration plots from these models were more closely around the diagonal line which indicated equality between the predicted and the true risks. It is noteworthy that, for the overall measures, difference between the frequentist model and the Bayesian model with informative priors was larger than difference among the Bayesian models with informative priors. Particularly between the Bayesian models with medium and highly informative priors, there is much less difference in the overall measures. Further, the frequentist models with expert opinion incorporated showed fairly similar patterns in results as the Bayesian models with informative priors.

**Table 2:** Results from the prediction models for data simulated with prevalence = 50%, ICC = .20, *n* = 5000 (J = S = 50, *n*_*j*_ = *n*_*c*_=100), *β*_1_ = 1.5 (the default setting)

	Optimal score	FREQ	BAYES.WI	BAYES.LI	BAYES.MI	BAYES.HI	FREQ.2	FREQ.3	FREQ.5
Overall Brier score	0	.191	.192	.179	.174	.170	.173	.170	.167
Overall C-index/AUC	1	.782	.781	.808	.818	.826	.822	.827	.833
Overall calibration slope	1	.911	.907	.957	.982	.989	.965	.972	.994
Within cluster C-index/AUC^a^	1	.805 [.037]	.805 [.037]	.805 [.037]	.805 [.037]	.805 [.037]	.805 [.037]	.805 [.037]	.805 [.037]
Within cluster calibration slope^a^	1	.914 [.102]	.914 [.102]	.947 [.091]	.956 [.078]	.963 [.058]	.954 [.092]	.973 [.080]	.977 [.062]

^a^mean[sd]

When it comes to cluster specific predictive performance, five models showed the same within cluster C-index means and variances, suggesting the same discriminative ability at the cluster level. This is because the random cluster effects only contribute to discrimination of subjects from different clusters. However, the Bayesian models with informative priors showed better within cluster calibration, as their within cluster calibration slopes were closer to 1 compared to the frequentist model. In addition, inclusion of more precise cluster level expert evidence led to smaller standard deviation for the within cluster calibration slopes.

**Fig. 3:**
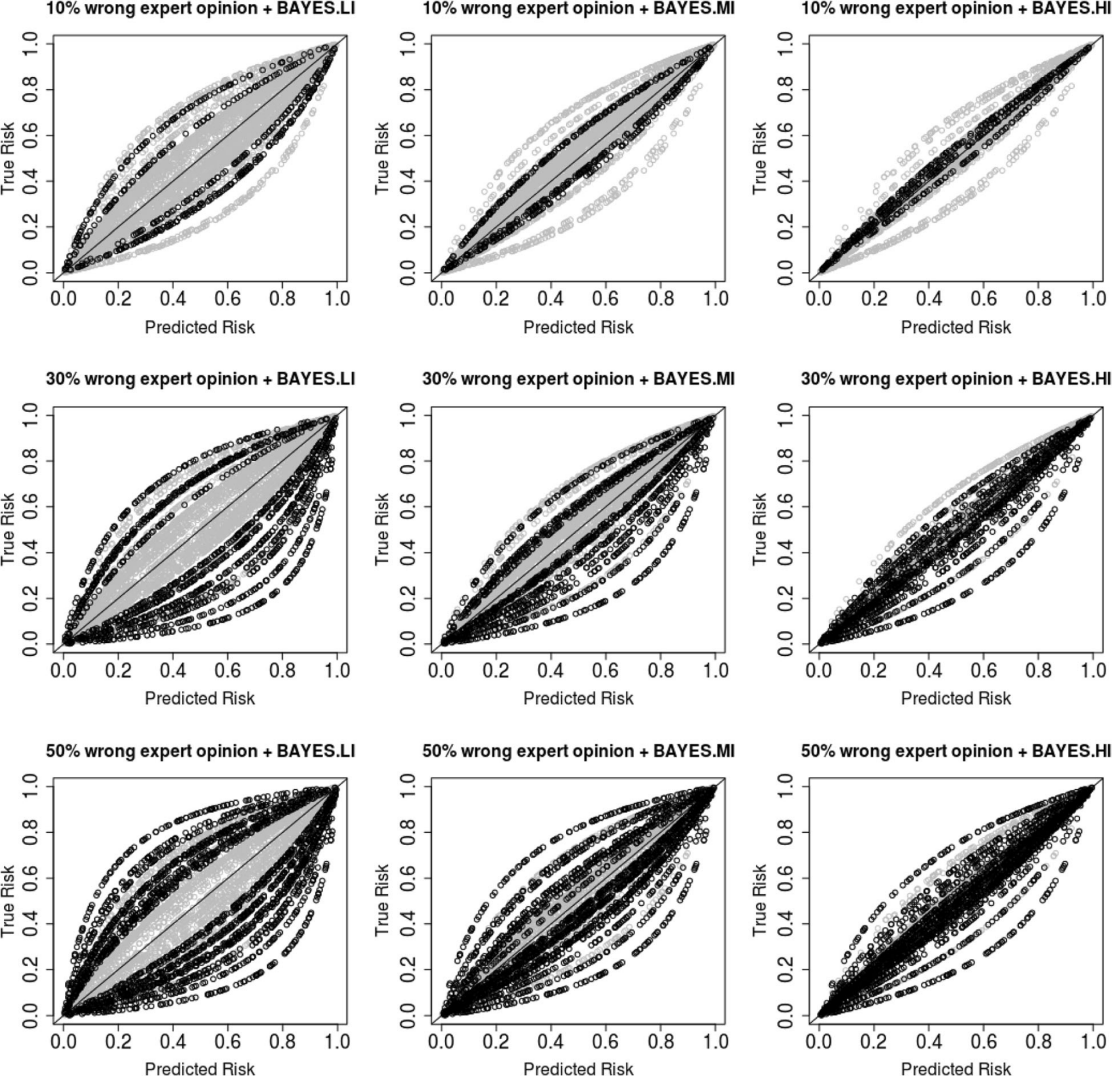
Calibration plots for Bayesian models using discrepant expert opinion as prior information for the random effects. Predicted risks are plotted against the true latent underlying risks for 5000 subjects from 50 equal sized clusters. Clusters using optimal expert opinion are displayed in grey color, whereas clusters using discrepant expert opinion are addressed in black color. The diagonal line is the line of identity (i.e., predicted risks are equal to the true risks). Each dot represents a subject, and each line formed by the dots represents a cluster

Further, as shown in Table 3, when the percentage of new clusters that incorporated discrepant expert opinion was 10%, all Bayesian models with informative priors still outperformed the frequentist model concerning the Brier score, the overall discrimination and the overall and within cluster calibration. When the number of clusters that incorporated discrepant expert opinion increased to 30%, the model with low informative priors performed similarly to the frequentist model, whereas the models with medium informative and highly informative priors still performed better. When the percentage was increased to 50%, the Bayesian model with low informative priors showed worse predictive performance than the frequentist model. However, the Bayesian models with medium and highly informative priors remained better in predictive performance. This phenomenon can also be seen in the 9 calibration plots in Fig. 3.

**Table 3:** Results from the Bayesian models with informative priors including different percentages of discrepant expert opinion

	FREQ	BAYES.WI	BAYES.LI			BAYES.MI			BAYES.HI		
Percentage wrong expert opinion	–	–	10%	30%	50%	10%	30%	50%	10%	30%	50%
Overall Brier score	.191	.192	.180	.192	.201	.174	.179	.182	.170	.173	.174
Overall C-index/AUC	.782	.781	.806	.781	.764	.818	.808	.801	.826	.821	.818
Overall calibration slope	.911	.907	.946	.874	.824	.982	.964	.950	.989	.988	.987
Within cluster C-index/AUC^a^	.805 [.037]	.805 [.037]	.805 [.037]	.805 [.037]	.805 [.037]	.805 [.037]	.805 [.037]	.805 [.037]	.805 [.037]	.805 [.037]	.805 [.037]
Within cluster calibration slope^a^	.914 [.102]	.914 [.102]	.946 [.091]	.939 [.100]	.935 [.100]	.953 [.077]	.939 [.084]	.935 [.085]	.962 [.059]	.953 [.068]	.951 [.070]

^a^mean[sd]

Multiple datasets for model development and prediction were generated using different seeds and results (not reported in the paper but available from the first author) showed the same structure among the prediction models. Furthermore, the eight sensitivity analyses showed the same patterns for the five models (see tables in Additional file 1: Appendix A), suggesting robustness of the Bayesian prediction models using informative priors. In addition, by varying the prevalence of the true binary outcomes, we noticed that it was more beneficial to add cluster specific priors to the random effects when the prevalence was closer to 50%. Results from different ICC values implied that in data with higher between cluster variance, it was more useful to include cluster specific expert opinion. In data with small cluster size and in data with small amount of clusters, the Bayesian models with cluster specific expert opinion still showed better performance than the frequentist model. Further, a weak predictor had negative impact on the frequentist model, adding cluster level informative priors in the Bayesian models showed clear improvement.

## Discussion

The simulation study showed that the Bayesian model with weakly informative priors performed similarly to the frequentist model where the random effect term was replaced with 0. It is hence possible to take the clustering structure from the new cluster(s) into account by means of keeping the random effects in the prediction model, without the loss of predictive ability. An additional benefit of using the Bayesian prediction models may be that they provide for each subject a distribution for the predicted risk. In many real world situations, such information may be preferred in comparison to point estimates.

Improvement was detected in results from models that had optimal cluster level expert opinion incorporated as informative priors for the random effects in the Bayesian approach as well as in the frequentist approach where expert opinion was incorporated as categorical variable. More specifically, these models showed better discrimination and calibration at the subject level, and better calibration within individual clusters. Comparison between these models also revealed that incorporation of more precisely specified expert knowledge would lead to better predictions. In addition, difference between the models without expert opinion and the models with low informative expert opinion (i.e., low informative prior for the Bayesian approach and a categorical variable with two levels for the frequentist approach) was larger than the difference among the models with cluster level expert opinion.

Results further revealed that the prediction model with low informative priors suffered the most from expert judgments that deviated from the true values of the random effects. When the percentage of clusters that include discrepant expert opinion as prior information exceeds 30%, the Bayesian model with low informative priors is not recommended. However, the other two Bayesian models with medium and highly informative priors seemed less influenced by incorporation of discrepant expert opinion and still gave better predictions compared to the frequentist model. This conclusion is however conditional on how the random effects distribution is divided and how discrepant expert opinion is defined. In this simulation study, we divided the random effects normal distribution into areas with equal proportions, hence intervals for the tail areas were much wider than the intervals at the center zone. We also assumed that when an expert provided information that deviated from the true value, she would select an adjacent area rather than select at random. Although it may perhaps be realistic to make such assumptions, we could as a consequence not fully investigate the impact of incorporating expert opinion that deviates from true values as prior information in prediction. Future studies may look further into this topic. Instead of only using the neighboring truncated areas, all other discrepant possibilities could be considered. In addition, the random effects distribution may be divided into equal intervals rather than equal areas.

To our knowledge, this study is the first attempt to combine model development data with expert opinion as prior information for random effects in prediction for new clusters. The simulated expert elicitation method is relatively novel as well. This method was also adapted and used in the frequentist models where expert opinion was incorporated as categorical variable in this study. It is nevertheless a simulation research, and only limited scenarios have been investigated.

Further, from a practical perspective, it may be a disadvantage and should be taken into account that the Bayesian prediction models proposed in this study are more time consuming than the frequentist prediction models.

Model evaluation was performed both at the subject and the cluster level. It is debatable which measures are more informative for prediction models [2]. In clinical practice, the within cluster measures might be most relevant. Other measures such as sensitivity and specificity which were not computed in this study could be used for model evaluation as well in real world applications.

## Conclusion

In the context of simulated data, we investigated prediction models which incorporated cluster level expert opinion in new clusters. Results showed that the prediction models with cluster level information were better at predicting the risks for the outcome in a new cluster than the commonly used frequentist model that replaced random effects with zero after model development. We focused on the Bayesian models we proposed, as it is more intuitive to use the Bayesian approach when incorporating prior knowledge into the analysis. Future research may focus on validation in real world data and evaluation of clinical benefits.

## Additional files

Additional file 1: **Appendix A.** Results for the sensitivity analyses. (DOCX 20 kb)

Additional file 2: **Appendix B.** R code for simulating the default setting data. (DOCX 14 kb)

## Abbreviations

BAYES.HI: Bayesian model with highly informative priors
BAYES.LI: Bayesian model with low informative priors
BAYES.MI: Bayesian model with medium informative priors
BAYES.WI: Bayesian model with weakly informative priors
FREQ: frequentist model
ICC: Intraclass correlation coefficient
MCMC: Markov chain Monte Carlo
ML: Maximum likelihood

## References

[1] Steyerberg EW (2009). Clinical prediction models; a practical approach to development, validation, and updating.

[2] Bouwmeester W, Twisk JWR, Kappen TH, Van Klei WL, Moons KGM, Vergouwe Y. Prediction models for clustered data: comparison of a random intercept and standard regression model. BMC Med Res Methodol. 2013; 10.1186/1471-2288-13-19. 10.1186/1471-2288-13-19 PMC3658967 23414436

[3] Hox JJ (2002). Multilevel analysis: techniques and applications.

[4] Bouwmeester W, Zuithoff NPA, Mallett S, Geerlings MI, Vergouwe Y, Steyerberg EW, Altman DG, Moons KGM. Reporting and methods in clinical prediction research: a systematic review. PLoS Med. 2012; 10.1371/journal.pmed.1001221. 10.1371/journal.pmed.1001221 PMC3358324 22629234

[5] Finkelman BS, French B, Kimmel SE. The prediction accuracy of dynamic mixed-effects models in clustered data. BioData Min. 2016; 10.1186/s13040-016-0084-6. 10.1186/s13040-016-0084-6 PMC4728760 26819631

[6] Van der Drift SG, Jorritsma R, Schonewille JT, Knijn HM, Stegeman JA (2012). Routine detection of hyperketonemia in dairy cows using Fourier transform infrared spectroscopy analysis of β-hydroxybutyrate and acetone in milk in combination with test-day information. J Dairy Sci.

[7] Spiegelhalter DJ, Abrams KR, Myles JP (2004). Bayesian approaches to clinical trials and health-care evaluation.

[8] O’Hagan A, Buck CE, Daneshkhah A, Eiser JR, Garthwaite PH, Jenkinson DJ, Oakley JE, Rakow T (2006). Uncertain Judgements: Eliciting Experts’ Probabilities.

[9] Bates D, Maechler M, Bolker B, Walker S, Christensen RHB, Singmann H, Dai B, Grothendieck G, Green P. Package 'lme4': linear mixed-effects models using 'Eigen' and S4. 2017; 19-04-2017. R CRAN project. Accessible via http://lme4.r-forge.r-project.org/. Ref Type: computer program.

[10] Ligges U, Sturtz S, Gelman A, Gorjanc G, Jackson C. Package ‘BRugs’: Interface to the 'OpenBUGS' MCMC software. 2017; 26-06-2017. R CRAN project. Accessible via https://CRAN.R-project.org/package=BRugs . Ref Type: computer program.

[11] Robert CP (1995). Simulation of truncated normal variables. Stat Comput.

[12] Van Calster B, Nieboer D, Vergouwe Y, De Cock B, Pencina MJ, Steyerberg EW (2016). A calibration hierarchy for risk models was defined: from utopia to empirical data. J Clin Epidemiol.

