# Supplementary material for: Prediction models for clustered data with informative priors for the random effects: a simulation study
Source: BMC Med Res Methodol. 2018 Aug 6;18:83. doi: 10.1186/s12874-018-0543-5 (PMC6080562; doi:10.1186/s12874-018-0543-5)
Supplement: Supplementary file 1 — Appendix A. Results for the sensitivity analyses. (DOCX 20 kb) [file 12874_2018_543_MOESM1_ESM.docx]

**Appendix A: Results for the sensitivity analyses**

**Table S1 Results from the five prediction models for data simulated with ICC**$\boldsymbol{=0.20}$**,** $\boldsymbol{n=5000}$ **(**$\boldsymbol{J=S=50}$**,** $\boldsymbol{n}_{\boldsymbol{j}}$ $\boldsymbol{=}$ $\boldsymbol{n}_{\boldsymbol{c}}\boldsymbol{=100}$**),** $\boldsymbol{\beta}_{\boldsymbol{1}}\boldsymbol{=1.5}$ **and varying prevalences**

|  |  | Prevalence = 10% | | | | |  | Prevalence = 25% | | | | |
| --- | --- | --- | --- | --- | --- | --- | --- | --- | --- | --- | --- | --- |
|  |  | FREQ | BAYES.WI | BAYES.LI | BAYES.MI | BAYES.HI |  | FREQ | BAYES.WI | BAYES.LI | BAYES.MI | BAYES.HI |
| Overall Brier score |  | .074 | .074 | .073 | .072 | .071 |  | .148 | .149 | .134 | .130 | .128 |
| Overall C-index / AUC |  | .810 | .812 | .825 | .833 | .841 |  | .789 | .788 | .836 | .845 | .851 |
| Overall calibration slope |  | .946 | .957 | .959 | .999 | 1.017 |  | .960 | .955 | 1.035 | 1.031 | 1.023 |
| Within cluster C-index / AUC * |  | .832 [.078] | .832 [.078] | .832 [.078] | .832 [.078] | .832 [.078] |  | .819 [.040] | .819 [.040] | .819 [.040] | .819 [.040] | .819 [.040] |
| Within cluster calibration slope * |  | .972 [.328] | .974 [.321] | .984 [.236] | .989 [.181] | .994 [.124] |  | .952 [.204] | .952 [.207] | 1.028 [.148] | 1.015 [.087] | 1.004 [.054] |

*mean[sd]

**Table S2 Results from the five prediction models for data simulated with prevalence** $\boldsymbol{=50\%}$**,** $\boldsymbol{n=5000}$ **(**$\boldsymbol{J=S=50}$**,** $\boldsymbol{n}_{\boldsymbol{j}}\boldsymbol{=}\boldsymbol{n}_{\boldsymbol{c}}\boldsymbol{=100}$**),** $\boldsymbol{\beta}_{\boldsymbol{1}}\boldsymbol{=}\boldsymbol{1.5}$ **and varying ICC values**

|  |  | ICC = 0.05 | | | | |  | ICC = 0.50 | | | | |
| --- | --- | --- | --- | --- | --- | --- | --- | --- | --- | --- | --- | --- |
|  |  | FREQ | BAYES.WI | BAYES.LI | BAYES.MI | BAYES.HI |  | FREQ | BAYES.WI | BAYES.LI | BAYES.MI | BAYES.HI |
| Overall Brier score |  | .178 | .178 | .175 | .174 | .173 |  | .199 | .198 | .167 | .157 | .153 |
| Overall C-index / AUC |  | .809 | .809 | .817 | .818 | .819 |  | .765 | .766 | .835 | .851 | .860 |
| Overall calibration slope |  | .970 | .970 | .983 | .986 | .987 |  | .834 | .839 | .946 | .985 | .1.006 |
| Within cluster C-index / AUC * |  | .815 [.045] | .815 [.045] | .815 [.045] | .815 [.045] | .815 [.045] |  | .814 [.044] | .814 [.044] | .814 [.044] | .814 [.044] | .814 [.044] |
| Within cluster calibration slope * |  | .970 [.022] | .970 [.022] | .979 [.019] | .981 [.017] | .982 [.013] |  | .815 [.208] | .819 [.208] | .934 [.213] | .952 [.182] | .961 [.144] |

*mean[sd]

**Table S3** **Results from the five prediction models for data simulated with prevalence** $\boldsymbol{=50\%}$**, ICC**$\boldsymbol{=0.20}$**,** $\boldsymbol{\beta}_{\boldsymbol{1}}\boldsymbol{=1.5}$ **and varying number of clusters (**$\boldsymbol{J, S}$**) or cluster size (**$\boldsymbol{n}_{\boldsymbol{j}}$**,** $\boldsymbol{n}_{\boldsymbol{c}}$**)**

|  |  | $n=2000 (J=S=20; n_{j}=n_{c}=100)$ | | | | |  | $n=1000 (J=S=50; n_{j}=n_{c}=20)$ | | | | |
| --- | --- | --- | --- | --- | --- | --- | --- | --- | --- | --- | --- | --- |
|  |  | FREQ | BAYES.WI | BAYES.LI | BAYES.MI | BAYES.HI |  | FREQ | BAYES.WI | BAYES.LI | BAYES.MI | BAYES.HI |
| Overall Brier score |  | .195 | .195 | .173 | .169 | .165 |  | .192 | .192 | .175 | .169 | .166 |
| Overall C-index / AUC |  | .774 | .775 | .821 | .830 | .837 |  | .783 | .783 | .817 | .827 | .835 |
| Overall calibration slope |  | .860 | .861 | .955 | .980 | .986 |  | .865 | .862 | .927 | .944 | .962 |
| Within cluster C-index / AUC* |  | .809 [.040] | .809 [.040] | .809 [.040] | .809 [.040] | .809 [.040] |  | .813 [.099] | .813 [.099] | .813 [.099] | .813 [.099] | .813 [.099] |
| Within cluster calibration slope* |  | .855 [.149] | .851 [.148] | .888 [.132] | .903 [.118] | .916 [.100] |  | .886 [.095] | .881 [.093] | .916 [.082] | .923 [.071] | .924 [.049] |

*mean[sd]

**Table S4** **Results from the five prediction models for data simulated with prevalence** $\boldsymbol{=50\%}$**, ICC**$\boldsymbol{=0.20}$**,** $\boldsymbol{n=5000}$ **(**$\boldsymbol{J=S=50}$**,** $\boldsymbol{n}_{\boldsymbol{j}}\boldsymbol{=}\boldsymbol{n}_{\boldsymbol{c}}\boldsymbol{=100}$**) and varying regression parameter values**

|  |  | $\beta_{1}$ = 0.5, Nagelkerke’s $R^{2}$ = .053 | | | | |  | $\beta_{1}$ = 3.0 , Nagelkerke’s $R^{2}$ = .596 | | | | |
| --- | --- | --- | --- | --- | --- | --- | --- | --- | --- | --- | --- | --- |
|  |  | FREQ | BAYES.WI | BAYES.LI | BAYES.MI | BAYES.HI |  | FREQ | BAYES.WI | BAYES.LI | BAYES.MI | BAYES.HI |
| Overall Brier score |  | .241 | .240 | .212 | .205 | .202 |  | .128 | .129 | .116 | .113 | .111 |
| Overall C-index / AUC |  | .613 | .618 | .725 | .747 | .753 |  | .900 | .899 | .917 | .921 | .923 |
| Overall calibration slope |  | .817 | .832 | 1.112 | 1.083 | 1.042 |  | .959 | .955 | .991 | .995 | 1.004 |
| Within cluster C-index / AUC * |  | .635 [.063] | .635 [.063] | .635 [.063] | .635 [.063] | .635 [.063] |  | .921 [.022] | .921 [.022] | .921 [.022] | .921 [.022] | .921 [.022] |
| Within cluster calibration slope * |  | .809 [.150] | .813 [.151] | .867 [.155] | .890 [.140] | .914 [.117] |  | .960 [.065] | .958 [.065] | .987 [.054] | .995 [.046] | .998 [.034] |

*mean[sd]
