# Supplementary material for: Prediction models for clustered data with informative priors for the random effects: a simulation study
Source: BMC Med Res Methodol. 2018 Aug 6;18:83. doi: 10.1186/s12874-018-0543-5 (PMC6080562; doi:10.1186/s12874-018-0543-5)
Supplement: Supplementary file 2 — Appendix B. R code for simulating the default setting data. (DOCX 14 kb) [file 12874_2018_543_MOESM2_ESM.docx]

**Appendix B: R code for simulating the default setting data**

while(TRUE){

########## determine the sample sizes ###############
J=50 # number of clusters per dataset
k=100 #number of subjects per cluster (equal size per cluster)

n_j <- rep(k, J)   # number of individuals in jth group
id <-seq(1,J)     
group <- rep(id,n_j) # group identifier per subject
N <- sum(n_j)

########## simulate datapoints for predictor X ##########
x1<-rnorm(N, 0,1) # development dataset

x<-rnorm(N,0,1) # predicton dataset

############## assign values to the parameters   #################
######## change beta_0 to adjust the prevalence ######
## prevalence=50%##
beta_0 <- 0.05

## prevalence= 25% ##
#beta_0 <- 1.5
## prevalence= 10% ##
#beta_0 <- -3

beta_1 <- 1.5

# intraclass correlation coefficient
icc <-0.20  # var(u_j) is about 0.822

# variance of the random intercept can be calculated based on ICC
var_u <- (icc*(pi^2)/3)/(1-icc)

u_j1 <- rnorm(J,0,sqrt(var_u)) # true random effects for development set
u_j <- rnorm(J,0,sqrt(var_u)) # true random effects for prediction set
#####################################################

# Now form the linear predictor
eta1 <- beta_0 + beta_1 * x1 + u_j1[group]

eta <- beta_0 + beta_1 * x + u_j[group]

# Transform back to the probability scale
p1 <- exp(eta1)/(1 + exp(eta1)) # true latent underyling risks for development set

p <- exp(eta)/(1+ exp(eta)) # true latent undelying risks for prediction set

# Binary outcomes sampled from Bernoulli distribution
y1 <- rbinom(N, 1, p1) # observed y's for development set
y <- rbinom(N, 1, p) # observed y's for prediction set

#######################################################
#### model development set and model prediction set
development.set <-data.frame(group,x1,y1)

prediction.set <- data.frame(group,x,y)

########### control the prevalence of the 2 datasets ########
prev1<-sum(y1)/(J*k)
prev<-sum(y)/(J*k)

#### sample data until both development and prediction sets have prevalence close to 50%
  if ((prev1>0.498 & prev1<0.502)&(prev>0.498 & prev<0.502)) break()
}
